# Supplementary material for: Association between the early use of beta-blocker and the risk of sepsis-associated acute kidney injury: A retrospective cohort study using the MIMIC-IV database
Source: PLoS One. 2025 Jun 16;20(6):e0325980. doi: 10.1371/journal.pone.0325980 (PMC12169561; doi:10.1371/journal.pone.0325980)
Supplement: S1 File — Table S2 The association between confounding variables and SA-AKI after PSM. Table S3 The association between confounding variables and SA-AKI before PSM. Table S4 The association between confounding variables and SA-AKI after PSM in early use of beta-blockers population. Figure 1A Distribution of propensity scores. Figure 1B Standardized mean differences before and after propensity score matching. (ZIP) [file pone.0325980.s001.zip › Supplementary information/Table S2 .docx]

Table S2 The association between confounding variables and SA-AKI after PSM

| Variables | Model 1 | | Model 2 | |
| --- | --- | --- | --- | --- |
|  | OR (95% CI) | *P* | OR (95% CI) | *P* |
| Age | 1.01 (1.00-1.02) | 0.007 | 1.02 (1.01-1.03) | <0.001 |
| Gender |  |  |  |  |
| Female | Ref |  |  |  |
| Male | 0.87 (0.70-1.07) | 0.183 |  |  |
| Race |  |  |  |  |
| Black | Ref |  |  |  |
| Others | 1.06 (0.66-1.70) | 0.801 |  |  |
| Unknown | 1.39 (0.84-2.30) | 0.195 |  |  |
| White | 1.19 (0.79-1.78) | 0.400 |  |  |
| Weight | 1.01 (1.01-1.02) | <0.001 | 1.01 (1.01-1.02) | <0.001 |
| Heart failure |  |  |  |  |
| No | Ref |  | Ref |  |
| Yes | 1.65 (1.27-2.16) | <0.001 | 1.31 (0.97-1.76) | 0.077 |
| AMI |  |  |  |  |
| No | Ref |  |  |  |
| Yes | 1.56 (1.05-2.31) | 0.026 |  |  |
| CKD |  |  |  |  |
| No | Ref |  |  |  |
| Yes | 1.09 (0.77-1.55) | 0.628 |  |  |
| Hypertension |  |  |  |  |
| No | Ref |  |  |  |
| Yes | 1.25 (1.00-1.56) | 0.047 |  |  |
| Diabetes |  |  |  |  |
| No | Ref |  |  |  |
| Yes | 1.23 (0.98-1.55) | 0.069 |  |  |
| Heart rate | 1.00 (1.00-1.01) | 0.088 |  |  |
| Systolic | 1.01 (1.00-1.01) | 0.018 | 1.01 (1.00-1.01) | 0.003 |
| Diastolic | 1.01 (1.00-1.01) | 0.122 |  |  |
| Respiratory rate | 1.02 (1.00-1.04) | 0.042 |  |  |
| Temperature | 1.17 (1.02-1.35) | 0.028 | 1.14 (0.97-1.33) | 0.108 |
| SpO_2_ | 0.95 (0.92-0.99) | 0.011 |  |  |
| SOFA | 1.24 (1.18-1.30) | <0.001 | 1.25 (1.19-1.31) | <0.001 |
| SAPS II | 1.03 (1.02-1.04) | <0.001 |  |  |
| CCI | 1.07 (1.02-1.12) | 0.007 |  |  |
| Creatinine | 1.18 (0.94-1.48) | 0.150 |  |  |
| BUN | 1.02 (1.01-1.03) | <0.001 | 1.02 (1.00-1.03) | 0.015 |
| Platelet | 1.00 (1.00-1.00) | 0.321 |  |  |
| WBC | 1.03 (1.01-1.05) | 0.001 | 1.03 (1.01-1.05) | 0.008 |
| RDW | 1.08 (1.03-1.14) | 0.003 |  |  |
| Hemoglobin | 1.02 (0.97-1.07) | 0.429 |  |  |
| Hematocrit | 1.01 (0.99-1.03) | 0.235 |  |  |
| Glucose | 1.00 (1.00-1.00) | 0.370 |  |  |
| Calcium | 1.08 (0.93-1.25) | 0.315 |  |  |
| Bicarbonate | 1.00 (0.97-1.03) | 0.985 |  |  |
| Sodium | 1.02 (0.99-1.04) | 0.146 |  |  |
| Potassium | 0.97 (0.85-1.11) | 0.635 |  |  |
| Chloride | 0.98 (0.96-1.00) | 0.042 | 0.98 (0.96-1.00) | 0.071 |
| INR | 1.15 (0.95-1.39) | 0.159 |  |  |
| PT | 1.01 (0.99-1.03) | 0.181 |  |  |
| PTT | 1.02 (1.00-1.03) | 0.041 |  |  |
| 24-hour urine-output | 1.00 (1.00-1.00) | 0.118 |  |  |
| Ventilation |  |  |  |  |
| No | Ref |  |  |  |
| Yes | 1.48 (1.02-2.13) | 0.037 |  |  |
| Vasopressor |  |  |  |  |
| No | Ref |  |  |  |
| Yes | 1.13 (0.92-1.40) | 0.256 |  |  |
| Loop diuretics |  |  |  |  |
| No | Ref |  | Ref |  |
| Yes | 1.38 (1.11-1.72) | 0.005 | 1.24 (0.97-1.59) | 0.093 |
| Nephrotoxic antibiotics |  |  |  |  |
| No | Ref |  |  |  |
| Yes | 1.12 (0.91-1.39) | 0.292 |  |  |
| CABG |  |  |  |  |
| No | Ref |  |  |  |
| Yes | 0.00 (0.00-Inf) | 0.972 |  |  |
| Insulin |  |  |  |  |
| No | Ref |  |  |  |
| Yes | 0.94 (0.76-1.16) | 0.555 |  |  |
| Platelet infusion |  |  |  |  |
| No | Ref |  |  |  |
| Yes | 1.28 (0.80-2.05) | 0.294 |  |  |
| eGFR | 1.00 (0.99-1.00) | 0.017 | 1.01 (1.00-1.02) | 0.002 |

SA-AKI, sepsis-associated acute kidney injury; .PSM, propensity score matching; OR, odds ratio; CI, confidence intervals; Ref, reference; AMI, acute myocardial infarction; CKD, chronic kidney disease; SpO_2_, saturation of peripheral oxygen; SOFA, sequential organ failure assessment; SAPS II, Simplified Acute Physiology Score II; CCI, Charlson comorbidity index; BUN, blood urea nitrogen; WBC, white blood cell; RDW, red cell distribution width; INR, international normalized ratio; PT, prothrombin time; PTT, partial thromboplastin time; CABG, coronary artery bypass grafting; eGFR, estimated glomerular filtration rate.

Model 1 adjusted for none. Model 2 adjusted for covariates screened after two-way stepwise regression.
