# Supplementary material for: Association between the early use of beta-blocker and the risk of sepsis-associated acute kidney injury: A retrospective cohort study using the MIMIC-IV database
Source: PLoS One. 2025 Jun 16;20(6):e0325980. doi: 10.1371/journal.pone.0325980 (PMC12169561; doi:10.1371/journal.pone.0325980)
Supplement: S1 File — Table S2 The association between confounding variables and SA-AKI after PSM. Table S3 The association between confounding variables and SA-AKI before PSM. Table S4 The association between confounding variables and SA-AKI after PSM in early use of beta-blockers population. Figure 1A Distribution of propensity scores. Figure 1B Standardized mean differences before and after propensity score matching. (ZIP) [file pone.0325980.s001.zip › Supplementary information/Table S1.docx]

Table S1 Sensitivity analysis before and after imputation

| Variables | Before imputation (N=4419) | After imputation (N=4419) | *P* |
| --- | --- | --- | --- |
| Weight, kg, Mean±SD | 78.12±18.36 | 78.03±17.23 | 0.830 |
| Heart rate, bpm, Mean±SD | 87.76±18.66 | 87.76±18.66 | 0.995 |
| Systolic, mmHg, Mean±SD | 123.36±23.29 | 123.36±23.29 | 1.000 |
| Diastolic, mmHg, Mean±SD | 67.08±15.65 | 67.08±15.64 | 0.996 |
| Respiratory rate, bpm, Mean±SD | 18.47±5.81 | 18.33±5.69 | 0.272 |
| Temperature, ℃, Mean±SD | 36.66±0.73 | 36.64±0.71 | 0.190 |
| SpO_2_, %, Mean±SD | 97.75±2.90 | 97.74±2.90 | 0.995 |
| BUN, mg/dL, M (Q₁, Q₃) | 17.00 (12.00, 25.00) | 17.00 (12.00, 25.00) | 0.817 |
| Platelet, K/uL, Mean±SD | 199.73±104.02 | 199.80±103.89 | 0.975 |
| WBC, K/uL, M (Q₁, Q₃) | 11.40 (8.20, 15.30) | 11.40 (8.30, 15.30) | 0.994 |
| RDW, %, Mean±SD | 14.69±2.16 | 14.69±2.16 | 0.998 |
| Hemoglobin, g/dL, Mean±SD | 10.62±2.21 | 10.62±2.21 | 0.993 |
| Hematocrit, %, Mean±SD | 31.94±6.49 | 31.94±6.49 | 0.999 |
| Glucose, mg/dL, (Q₁, Q₃) | 129.00 (107.00, 160.00) | 129.00 (107.00, 160.00) | 0.997 |
| Calcium, mmol/L, Mean±SD | 8.27±0.81 | 8.25±0.75 | 0.230 |
| Bicarbonate, mEq/L, Mean±SD | 23.60±4.18 | 23.60±4.18 | 0.997 |
| Sodium, mEq/L, Mean±SD | 137.86±4.71 | 137.86±4.70 | 1.000 |
| Potassium, mEq/L, Mean±SD | 4.24±0.77 | 4.24±0.77 | 0.992 |
| Chloride, mEq/L, Mean±SD | 104.84±5.87 | 104.84±5.87 | 0.999 |
| INR, M (Q₁, Q₃) | 1.30 (1.10, 1.50) | 1.29 (1.10, 1.40) | 0.581 |
| PT, second, M (Q₁, Q₃) | 14.30 (12.70, 16.20) | 14.35 (12.90, 16.00) | 0.713 |
| PTT, second, Mean±SD | 32.41±7.72 | 32.42±7.38 | 0.967 |
| 24-hour urine-output, mL, M (Q₁, Q₃) | 2275.00 (1665.00, 3045.00) | 2275.00 (1665.00, 3040.00) | 0.962 |

SD, standard deviation; M (Q_1_, Q_3_), median (first Quartile, third Quartile); SpO_2_, saturation of peripheral oxygen; BUN, blood urea nitrogen; WBC, white blood cell; RDW, red cell distribution width; INR, international normalized ratio; PT, prothrombin time; PTT, partial thromboplastin time.
