# Supplementary material for: Association between the early use of beta-blocker and the risk of sepsis-associated acute kidney injury: A retrospective cohort study using the MIMIC-IV database
Source: PLoS One. 2025 Jun 16;20(6):e0325980. doi: 10.1371/journal.pone.0325980 (PMC12169561; doi:10.1371/journal.pone.0325980)
Supplement: S1 File — Table S2 The association between confounding variables and SA-AKI after PSM. Table S3 The association between confounding variables and SA-AKI before PSM. Table S4 The association between confounding variables and SA-AKI after PSM in early use of beta-blockers population. Figure 1A Distribution of propensity scores. Figure 1B Standardized mean differences before and after propensity score matching. (ZIP) [file pone.0325980.s001.zip › Supplementary information/Table S4 .docx]

Table S4 The association between confounding variables and SA-AKI after PSM in early use of beta-blockers population

| Variables | Model 1 | | Model 2 | |
| --- | --- | --- | --- | --- |
|  | OR (95% CI) | *P* | OR (95% CI) | *P* |
| Age | 1.02 (1.01-1.03) | 0.001 | 1.02 (1.01-1.04) | 0.001 |
| Gender |  |  |  |  |
| Female | Ref |  |  |  |
| Male | 0.72 (0.54-0.97) | 0.034 |  |  |
| Race |  |  |  |  |
| Black | Ref |  |  |  |
| Others | 1.33 (0.69-2.57) | 0.389 |  |  |
| Unknown | 1.59 (0.79-3.19) | 0.197 |  |  |
| White | 1.55 (0.88-2.74) | 0.129 |  |  |
| Weight | 1.01 (1.00-1.02) | 0.008 | 1.01 (1.00-1.02) | 0.015 |
| Heart failure |  |  |  |  |
| No | Ref |  | Ref |  |
| Yes | 1.95 (1.35-2.83) | <0.001 | 1.42 (0.93-2.17) | 0.103 |
| AMI |  |  |  |  |
| No | Ref |  |  |  |
| Yes | 2.19 (1.25-3.82) | 0.006 |  |  |
| CKD |  |  |  |  |
| No | Ref |  |  |  |
| Yes | 1.19 (0.72-1.95) | 0.503 |  |  |
| Hypertension |  |  |  |  |
| No | Ref |  |  |  |
| Yes | 1.11 (0.82-1.52) | 0.498 |  |  |
| Diabetes |  |  |  |  |
| No | Ref |  |  |  |
| Yes | 1.30 (0.94-1.78) | 0.111 |  |  |
| Heart rate | 1.01 (1.00-1.02) | 0.008 |  |  |
| Systolic | 1.01 (1.00-1.01) | 0.022 | 1.01 (1.00-1.02) | 0.010 |
| Diastolic | 1.01 (1.00-1.02) | 0.088 |  |  |
| Respiratory rate | 1.04 (1.01-1.07) | 0.002 |  |  |
| Temperature | 1.45 (1.17-1.79) | 0.001 | 1.35 (1.06-1.72) | 0.016 |
| SpO_2_ | 0.89 (0.84-0.94) | <0.001 |  |  |
| SOFA | 1.26 (1.18-1.35) | <0.001 | 1.28 (1.19-1.38) | <0.001 |
| SAPS II | 1.03 (1.02-1.05) | <0.001 |  |  |
| Charlson comorbidity index | 1.11 (1.04-1.18) | 0.003 |  |  |
| Creatinine | 1.35 (0.97-1.90) | 0.077 |  |  |
| BUN | 1.03 (1.02-1.05) | <0.001 | 1.03 (1.01-1.05) | 0.011 |
| Platelet | 1.00 (1.00-1.00) | 0.081 |  |  |
| WBC | 1.03 (1.00-1.06) | 0.023 | 1.03 (1.00-1.06) | 0.070 |
| RDW | 1.24 (1.14-1.35) | <0.001 |  |  |
| Hemoglobin | 0.99 (0.93-1.06) | 0.866 |  |  |
| Hematocrit | 1.00 (0.98-1.03) | 0.706 |  |  |
| Glucose | 1.00 (1.00-1.00) | 0.800 |  |  |
| Calcium | 1.14 (0.92-1.42) | 0.237 |  |  |
| Bicarbonate | 1.02 (0.98-1.06) | 0.424 |  |  |
| Sodium | 1.03 (1.00-1.07) | 0.084 |  |  |
| Potassium | 0.94 (0.77-1.14) | 0.510 |  |  |
| Chloride | 0.96 (0.93-0.99) | 0.009 | 0.97 (0.93-1.00) | 0.041 |
| INR | 1.08 (0.85-1.37) | 0.553 |  |  |
| PT | 1.01 (0.98-1.03) | 0.598 |  |  |
| PTT | 1.01 (0.99-1.03) | 0.229 |  |  |
| 24-hour urine-output | 1.00 (1.00-1.00) | 0.019 |  |  |
| Ventilation |  |  |  |  |
| No | Ref |  |  |  |
| Yes | 1.50 (0.88-2.57) | 0.137 |  |  |
| Vasopressor |  |  |  |  |
| No | Ref |  |  |  |
| Yes | 0.94 (0.69-1.27) | 0.675 |  |  |
| Loop diuretics |  |  |  |  |
| No | Ref |  | Ref |  |
| Yes | 1.32 (0.96-1.81) | 0.084 | 1.14 (0.79-1.64) | 0.486 |
| Nephrotoxic antibiotics |  |  |  |  |
| No | Ref |  |  |  |
| Yes | 1.17 (0.86-1.59) | 0.313 |  |  |
| CABG |  |  |  |  |
| No | Ref |  |  |  |
| Yes | 0.00 (0.00-Inf) | 0.972 |  |  |
| Insulin |  |  |  |  |
| No | Ref |  |  |  |
| Yes | 0.71 (0.52-0.95) | 0.023 |  |  |
| Platelet infusion |  |  |  |  |
| No | Ref |  |  |  |
| Yes | 0.73 (0.37-1.45) | 0.372 |  |  |
| eGFR | 0.99 (0.98-1.00) | 0.001 | 1.01 (1.00-1.02) | 0.033 |

SA-AKI, sepsis-associated acute kidney injury; .PSM, propensity score matching; OR, odds ratio; CI, confidence intervals; Ref, reference; AMI, acute myocardial infarction; CKD, chronic kidney disease; SpO_2_, saturation of peripheral oxygen; SOFA, sequential organ failure assessment; SAPS II, Simplified Acute Physiology Score II; CCI, Charlson comorbidity index; BUN, blood urea nitrogen; WBC, white blood cell; RDW, red cell distribution width; INR, international normalized ratio; PT, prothrombin time; PTT, partial thromboplastin time; CABG, coronary artery bypass grafting; eGFR, estimated glomerular filtration rate.

Model 1 adjusted for none. Model 2 adjusted for covariates screened after two-way stepwise regression.
