# Supplementary material for: Association between the early use of beta-blocker and the risk of sepsis-associated acute kidney injury: A retrospective cohort study using the MIMIC-IV database
Source: PLoS One. 2025 Jun 16;20(6):e0325980. doi: 10.1371/journal.pone.0325980 (PMC12169561; doi:10.1371/journal.pone.0325980)
Supplement: S1 File — Table S2 The association between confounding variables and SA-AKI after PSM. Table S3 The association between confounding variables and SA-AKI before PSM. Table S4 The association between confounding variables and SA-AKI after PSM in early use of beta-blockers population. Figure 1A Distribution of propensity scores. Figure 1B Standardized mean differences before and after propensity score matching. (ZIP) [file pone.0325980.s001.zip › Supplementary information/Table S3 .docx]

Table S3 The association between confounding variables and SA-AKI before PSM

| Variables | Model 1 | | Model 2 | |
| --- | --- | --- | --- | --- |
|  | OR (95% CI) | *P* | OR (95% CI) | *P* |
| Age | 1.01 (1.01-1.02) | <0.001 | 1.01 (1.00-1.01) | 0.003 |
| Gender |  |  |  |  |
| Female | Ref |  |  |  |
| Male | 1.02 (0.90-1.15) | 0.807 |  |  |
| Race |  |  |  |  |
| Black | Ref |  |  |  |
| Others | 1.02 (0.78-1.35) | 0.867 |  |  |
| Unknown | 1.20 (0.90-1.59) | 0.217 |  |  |
| White | 1.12 (0.88-1.43) | 0.363 |  |  |
| Weight | 1.01 (1.01-1.02) | <0.001 | 1.02 (1.01-1.02) | <0.001 |
| Heart failure |  |  |  |  |
| No | Ref |  | Ref |  |
| Yes | 1.89 (1.64-2.18) | <0.001 | 1.36 (1.15-1.60) | <0.001 |
| AMI |  |  |  |  |
| No | Ref |  | Ref |  |
| Yes | 1.83 (1.49-2.25) | <0.001 | 1.30 (1.03-1.63) | 0.024 |
| CKD |  |  |  |  |
| No | Ref |  |  |  |
| Yes | 1.17 (0.98-1.40) | 0.075 |  |  |
| Hypertension |  |  |  |  |
| No | Ref |  |  |  |
| Yes | 1.24 (1.10-1.40) | <0.001 |  |  |
| Diabetes |  |  |  |  |
| No | Ref |  |  |  |
| Yes | 1.30 (1.13-1.48) | <0.001 |  |  |
| Heart rate | 1.00 (1.00-1.01) | 0.106 |  |  |
| Systolic | 1.00 (1.00-1.00) | 0.186 |  |  |
| Diastolic | 1.00 (1.00-1.01) | 0.351 |  |  |
| Respiratory rate | 1.02 (1.01-1.04) | <0.001 | 1.02 (1.01-1.03) | 0.004 |
| Temperature | 1.06 (0.97-1.15) | 0.186 |  |  |
| SpO_2_ | 0.96 (0.94-0.98) | <0.001 |  |  |
| SOFA | 1.23 (1.20-1.26) | <0.001 | 1.21 (1.17-1.24) | <0.001 |
| SAPS II | 1.03 (1.03-1.04) | <0.001 | 1.01 (1.00-1.01) | 0.055 |
| Charlson comorbidity index | 1.08 (1.06-1.11) | <0.001 |  |  |
| Creatinine | 1.28 (1.15-1.43) | <0.001 | 0.81 (0.71-0.92) | 0.001 |
| BUN | 1.01 (1.01-1.02) | <0.001 |  |  |
| Platelet | 1.00 (1.00-1.00) | 0.181 |  |  |
| WBC | 1.01 (1.00-1.02) | 0.005 | 1.01 (1.00-1.01) | 0.153 |
| RDW | 1.04 (1.01-1.07) | 0.003 |  |  |
| Hemoglobin | 1.01 (0.99-1.04) | 0.333 |  |  |
| Hematocrit | 1.01 (1.00-1.02) | 0.056 |  |  |
| Glucose | 1.00 (1.00-1.00) | 0.002 |  |  |
| Calcium | 1.06 (0.98-1.15) | 0.152 |  |  |
| Bicarbonate | 1.00 (0.99-1.02) | 0.524 |  |  |
| Sodium | 1.00 (0.99-1.01) | 0.797 |  |  |
| Potassium | 1.03 (0.96-1.11) | 0.428 |  |  |
| Chloride | 0.98 (0.97-0.99) | <0.001 | 0.98 (0.97-0.99) | 0.001 |
| INR | 1.27 (1.13-1.42) | <0.001 |  |  |
| PT | 1.02 (1.01-1.03) | 0.001 |  |  |
| PTT | 1.03 (1.02-1.04) | <0.001 | 1.01 (1.00-1.02) | 0.004 |
| 24-hour urine-output | 1.00 (1.00-1.00) | 0.020 | 1.00 (1.00-1.00) | <0.001 |
| Ventilation |  |  |  |  |
| No | Ref |  | Ref |  |
| Yes | 1.88 (1.57-2.26) | <0.001 | 1.42 (1.17-1.73) | <0.001 |
| Vasopressor |  |  |  |  |
| No | Ref |  |  |  |
| Yes | 1.57 (1.39-1.77) | <0.001 |  |  |
| Loop diuretics |  |  |  |  |
| No | Ref |  | Ref |  |
| Yes | 1.55 (1.35-1.77) | <0.001 | 1.18 (1.01-1.38) | 0.032 |
| Nephrotoxic antibiotics |  |  |  |  |
| No | Ref |  | Ref |  |
| Yes | 1.14 (1.01-1.28) | 0.039 | 0.83 (0.73-0.95) | 0.008 |
| CABG |  |  |  |  |
| No | Ref |  | Ref |  |
| Yes | 5.24 (2.00-13.77) | 0.001 | 4.10 (1.52-11.09) | 0.005 |
| Insulin |  |  |  |  |
| No | Ref |  |  |  |
| Yes | 1.13 (1.01-1.28) | 0.036 |  |  |
| Platelet infusion |  |  |  |  |
| No | Ref |  |  |  |
| Yes | 1.21 (0.93-1.58) | 0.153 |  |  |
| eGFR | 0.99 (0.99-0.99) | <0.001 |  |  |

SA-AKI, sepsis-associated acute kidney injury; .PSM, propensity score matching; OR, odds ratio; CI, confidence intervals; Ref, reference; AMI, acute myocardial infarction; CKD, chronic kidney disease; SpO_2_, saturation of peripheral oxygen; SOFA, sequential organ failure assessment; SAPS II, Simplified Acute Physiology Score II; CCI, Charlson comorbidity index; BUN, blood urea nitrogen; WBC, white blood cell; RDW, red cell distribution width; INR, international normalized ratio; PT, prothrombin time; PTT, partial thromboplastin time; CABG, coronary artery bypass grafting; eGFR, estimated glomerular filtration rate.

Model 1 adjusted for none. Model 2 adjusted for covariates screened after two-way stepwise regression.
