# Supplementary figures and images for: Association between the early use of beta-blocker and the risk of sepsis-associated acute kidney injury: A retrospective cohort study using the MIMIC-IV database
Source: PLoS One. 2025 Jun 16;20(6):e0325980. doi: 10.1371/journal.pone.0325980 (PMC12169561; doi:10.1371/journal.pone.0325980)

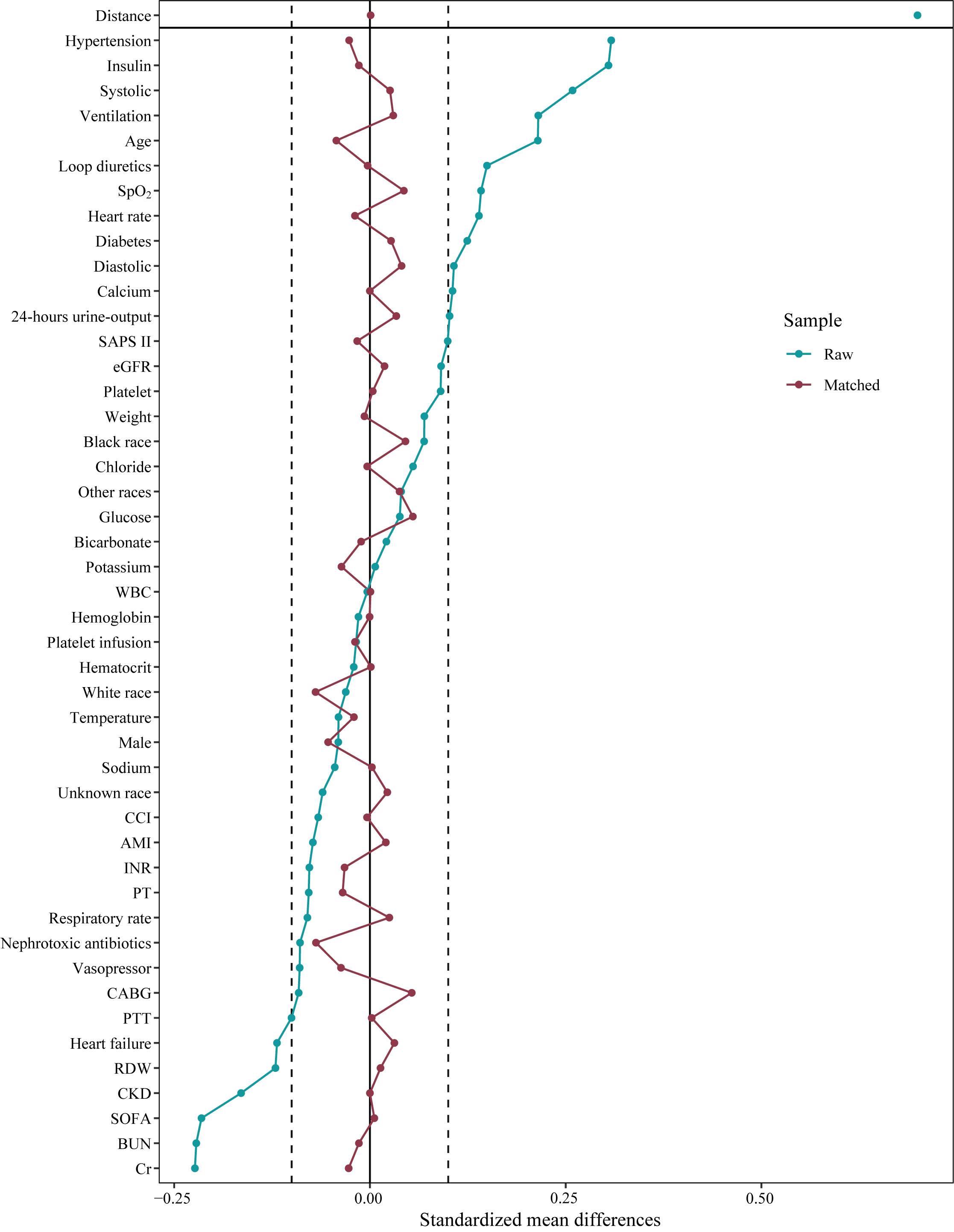

Supplement: S1 File — Table S2 The association between confounding variables and SA-AKI after PSM. Table S3 The association between confounding variables and SA-AKI before PSM. Table S4 The association between confounding variables and SA-AKI after PSM in early use of beta-blockers population. Figure 1A Distribution of propensity scores. Figure 1B Standardized mean differences before and after propensity score matching. (ZIP) [file pone.0325980.s001.zip › Supplementary information/Supplementary Figure 1B.tif]

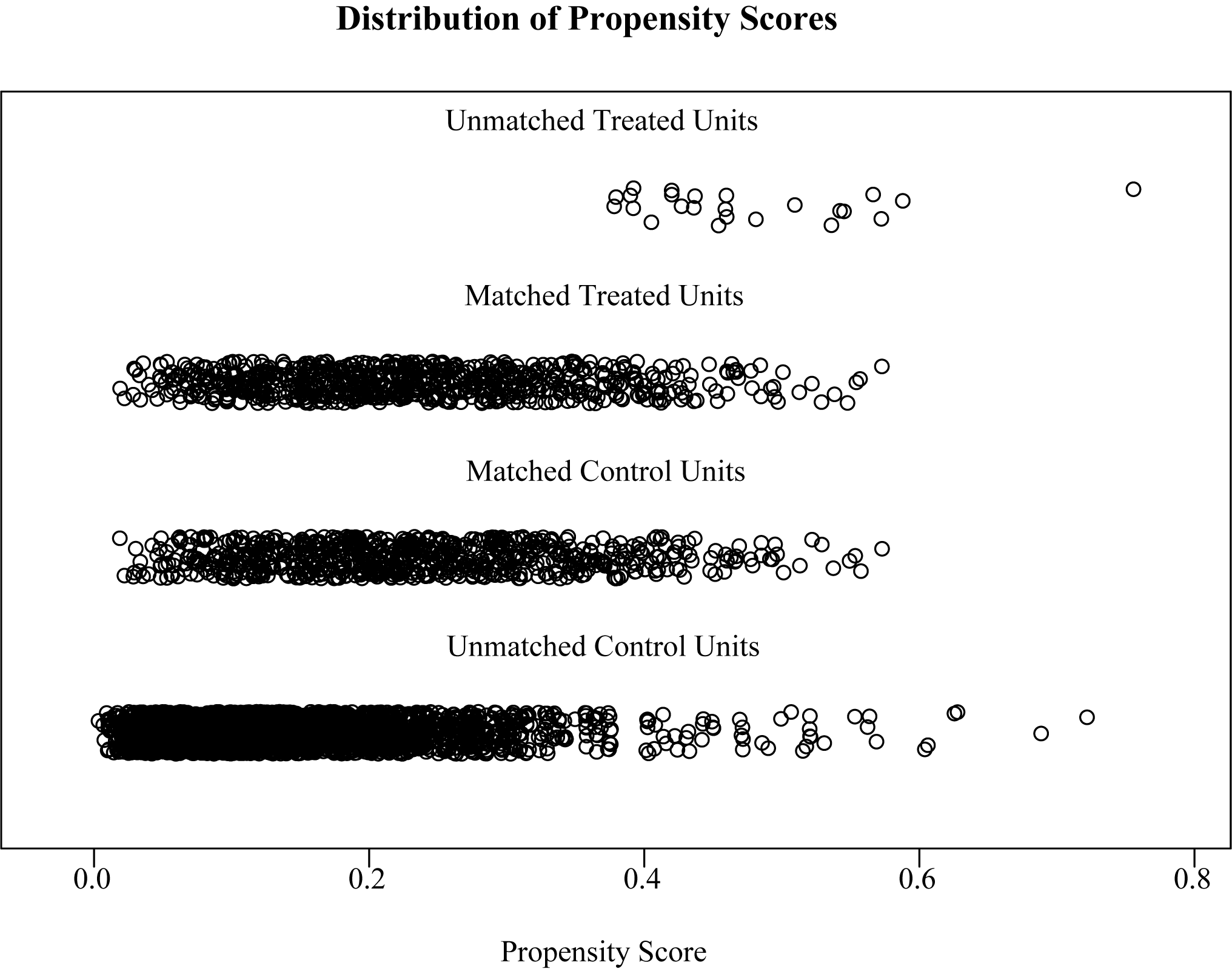

Supplement: S1 File — Table S2 The association between confounding variables and SA-AKI after PSM. Table S3 The association between confounding variables and SA-AKI before PSM. Table S4 The association between confounding variables and SA-AKI after PSM in early use of beta-blockers population. Figure 1A Distribution of propensity scores. Figure 1B Standardized mean differences before and after propensity score matching. (ZIP) [file pone.0325980.s001.zip › Supplementary information/Supplementary Figure 1A.tif]
